# A Histomorphologic and Ultrastructural Report of an Oncocytic Adrenal Cortical Carcinoma With Clinicoradiologic Correlates

**DOI:** 10.7759/cureus.74184

**Published:** 2024-11-21

**Authors:** Christine D Santos, Dennis Jose S Carbonell

**Affiliations:** 1 Department of Pathology and Laboratory Medicine, National Kidney and Transplant Institute, Quezon City, PHL

**Keywords:** adrenal gland neoplasms, adrenocortical carcinoma (acc), electron microscopy, genito-urinary pathology, immunohistochemistry(ihc), lin-weiss-bisceglia criteria, mitochondria, oncocytic adrenocortical neoplasms, oncocytic variant

## Abstract

Oncocytic adrenal cortical carcinoma (ACC) is a rare malignant adrenal cortical tumor with limited documented case reports. Herein, a 65-year-old female patient presented with a large, solid adrenal mass. A diagnosis of oncocytic ACC was rendered with the following tumor characteristics: The tumor entirely consists of diffuse sheets of polygonal cells with bizarre nuclear atypia and deeply eosinophilic cytoplasm. The panel of immunohistochemistry revealed its adrenocortical nature, with synaptophysin and melan-A expression and the corresponding negativity of pancytokeratin and chromogranin. Moreover, an ultrastructural demonstration of the oncocytic nature of the mass is also presented.

## Introduction

Adrenal cortical carcinoma (ACC) is the culprit of most deaths among primary adrenal tumors. The prognosis is poor, with a five-year overall survival rate of 37-47% [1]. The Surveillance, Epidemiology, and End Results (SEER) Program reports an annual incidence of 1.02 cases per one million in the years 1973 to 2012 [2]. ACC has multiple histologic variants: oncocytic ACC, myxoid ACC, and sarcomatoid ACC [1]. With about 30-40 cases documented in the literature, the oncocytic variant is the most prevalent of them, second only to conventional ACC. Fortunately, compared to conventional ACC, the prognosis is twice as favorable.

Oncocytes are epithelial cells characterized by an abundant, highly eosinophilic cytoplasm with increased mitochondria. In the oncocytic adrenal cortical neoplasms (OANs), these are further subclassified as oncocytic adrenocortical adenoma (oncocytoma) in 35% of cases, oncocytic adrenocortical neoplasm of uncertain malignant potential in 41% of cases, and oncocytic ACC in 24% of cases [2]. The World Health Organization (WHO) recommends using the Lin-Weiss-Bisceglia (LWB) criteria, which identifies a malignant oncocytic adrenal neoplasm with at least one of three major criteria fulfilled [1].

## Case presentation

**Figure 1:**
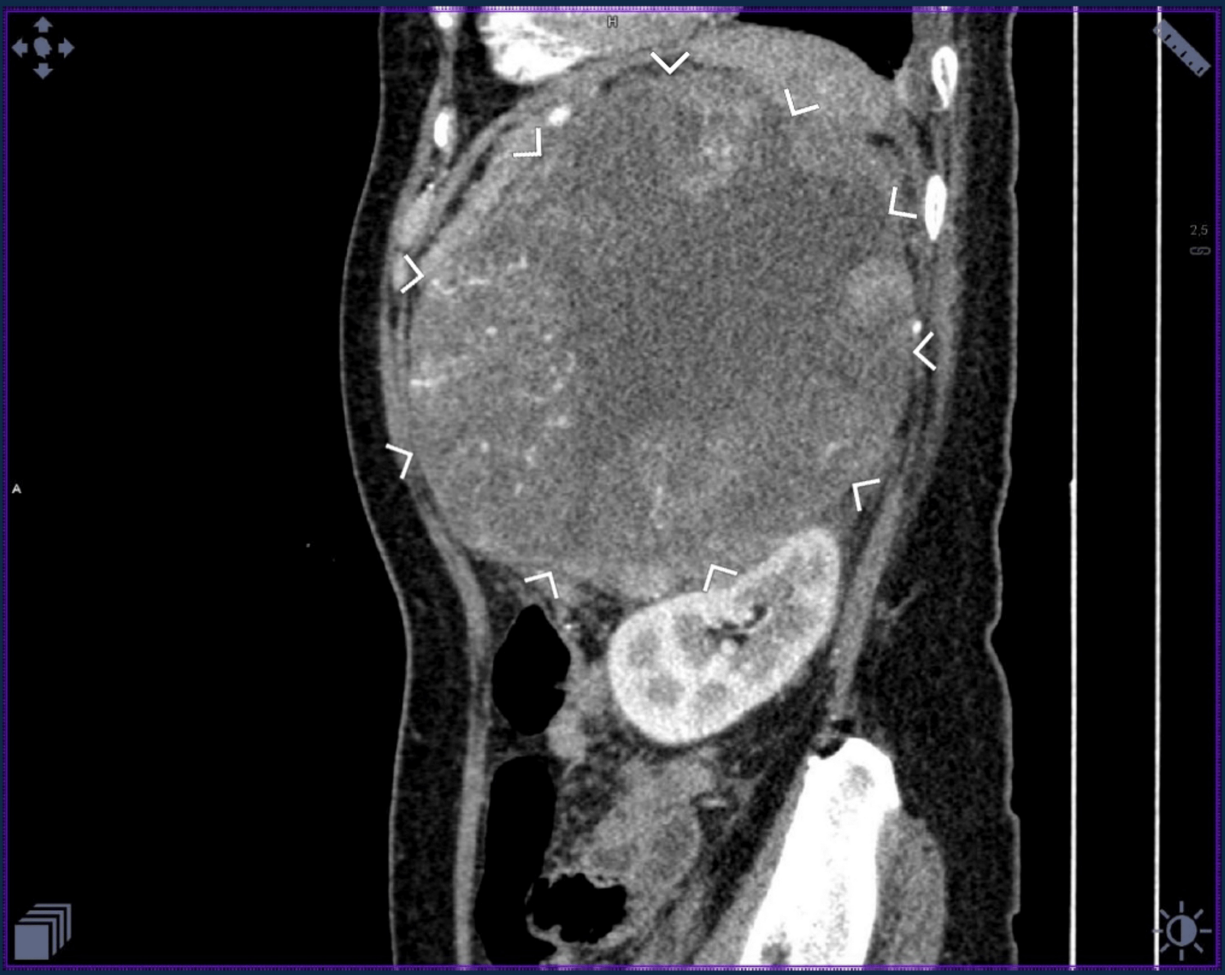
Computed tomography urogram, sagittal view, showing the large adrenal mass.

The case is a 65-year-old female with a three-month history of boring epigastric pain radiating to the periumbilical area, with associated nausea, vomiting, and bloatedness. These were not relieved by antacids. A computed tomography (CT) urogram revealed a large, lobulated, heterogeneously enhancing mass measuring 14.2 cm × 11.8 cm × 12.5 cm in the left upper hemiabdomen (Figure 1). Hormonal screening parameters revealed normal serum aldosterone, cortisol, fasting blood sugar, electrolytes, and kidney function tests, and normal urine vanillylmandelic acid, metanephrine, normetanephrine, and total metanephrine (Table 1). The clinical impression was a non-functioning high-grade adrenal carcinoma.

**Table 1:** Results of the blood and urine ancillary studies of patient.

Analyte	Result	Reference values
Serum blood urea nitrogen (BUN)	11 mg/dL	9–23 mg/dL
Serum creatinine	0.63 mg/dL	0.5–0.8 mg/dL
Serum sodium	142 mmol/L	136–145 mmol/L
Serum potassium	5.1 mmol/L	3.5–5.1 mmol/L
Serum calcium	8.6 mg/dL	8.4–10.2 mg/dL
Serum magnesium	2.46 mg/dL	1.8–3.0 mg/dL
Serum albumin	4.01 g/dL	3.8–5.0 g/dL
Fasting plasma glucose	89 mg/dL	74–99 mg/dL
Serum aldosterone	173 pg/mL	67–335 pg/mL
Serum morning cortisol	288.9 nmol/L	172–497 nmol/L
Urine vanillylmandelic acid	6.27 mcg/ 24 h	0.6–13.6 mcg/ 24h
Urine metanephrine	67 mcg/ 24 h	24–96 mcg/ 24 h
Urine normetanephrine	155 mcg/ 24h	75–375 mcg/ 24 h
Urine total metanephrine	222 mcg/ 24 h	<300 mcg/24 h

**Figure 2:**
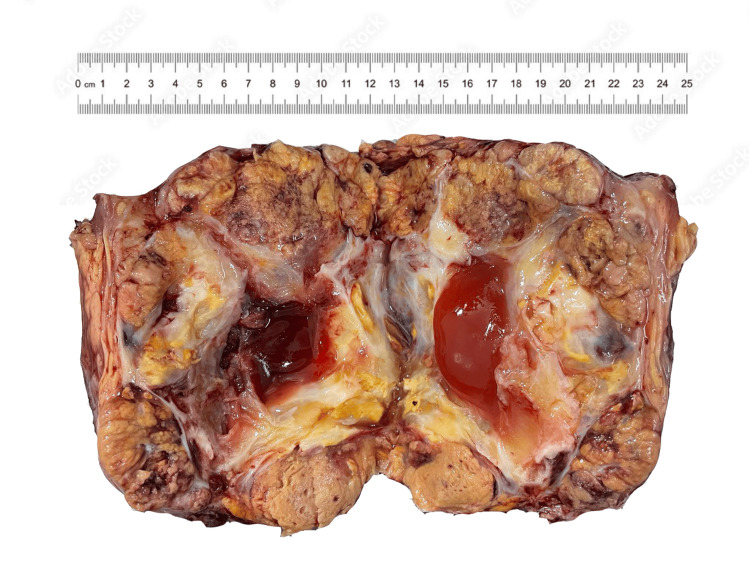
Gross appearance of adrenal mass.

A thoracoabdominal adrenalectomy was performed with good planes of demarcation between the mass and the adjacent structures. The adrenal gland is 16 cm × 14 cm × 8.3 cm, and 1,250 g. There are surrounding scant adipose tissues, and no distinct normal parenchymal tissues were identified. The mass is ovoid to irregular, circumscribed, heterogenous, tan-yellow, variegated, and soft to firm, with several pale red materials and multiple foci of hemorrhages and necrosis (Figure 2).

**Figure 3:**
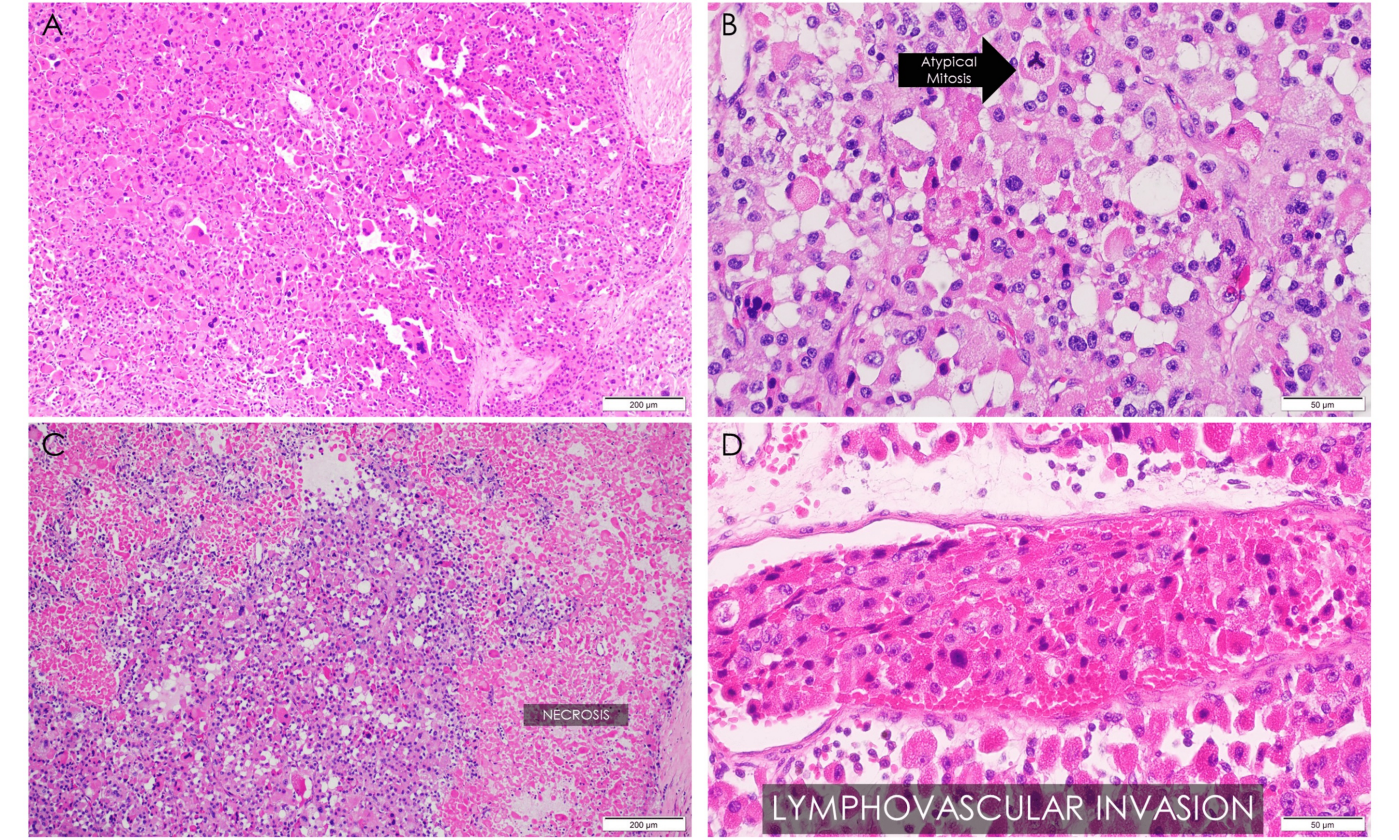
Hematoxylin and eosin-stained histologic section of oncocytic ACC. (A) Low power magnification (×100); (B) high-power magnification with atypical mitosis (×400); (C) low-power magnification showing geographic necrosis (×100); (D) high-power magnification with lymphovascular invasion (×400).

**Figure 4:**
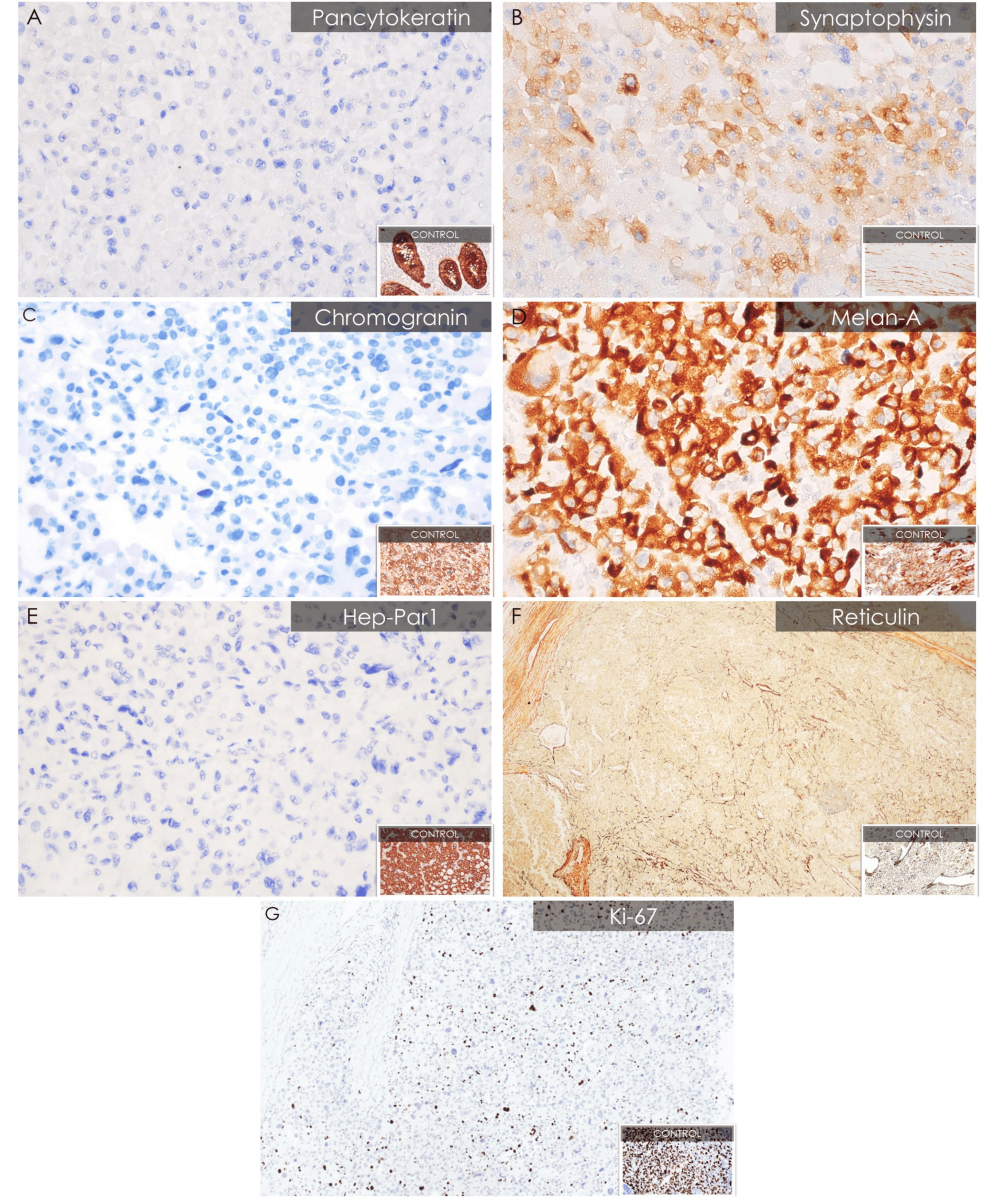
Immunohistochemical studies and special stain of oncocytic ACC. (A) Pancytokeratin (×400); (B) synaptophysin (×400); (C) chromogranin (×100); (D) Melan-A (×400); (E) Hep-Par1 (×400); (F) reticulin (×100); (G) Ki-67 (×400).

Histologic sections show the tumor in sheets and alveolar nests separated by thin fibrous septate (Figure 3A). The entire tumor shows these polygonal cells with abundant, deeply eosinophilic cytoplasm. The neoplastic cells have round to severely pleomorphic, hyperchromatic nuclei with coarse chromatin and one to multiple nucleoli (Figure 3B). The mitotic rate is 10 to 15 per 50 high power fields (hpf) and with atypical forms (Figure 3B). Geographic areas of necrosis (Figure 3C) and venous structure invasion were found (Figure 3D). The immunohistochemistry profile of the mass confirms the presence of an adrenal cortical tumor, exhibiting positive expression of synaptophysin (Figure 4B) and Melan-A (Figure 4D), and negative expression of pancytokeratin (Figure 4A), chromogranin (Figure 4C), and hep-Par1 (Figure 4E). There is also a loss of the network of reticulin fibers, which normally encircles the nests of cells, depicting an abnormal reticulin pattern (Figure 4F).

Oncocytic adrenal neoplasms are evaluated using one of the following systems: Lin-Weiss Bisceglia (LWB), the Helsinki scoring system, and the reticulin algorithm [1]. All three major and all four minor LWB criteria (Table 2) were satisfied, classifying this case as an oncocytic ACC. The Helsinki scoring system (Table 3) acquired a total score of 25, indicating an adverse prognosis, with a mitotic rate of at least ten per 50 hpf (score = 3), tumor necrosis (score = 5), and a Ki-67 of 10-15% (score = at least 10). Lastly, the altered reticulin framework in association with an increased mitotic rate, the presence of tumor necrosis, and vascular invasion also fulfill the criteria for malignancy in the reticulin algorithm (Table 4).

**Table 2:** Lin-Weiss-Bisceglia criteria for oncocytic adrenal neoplasms. Malignant: at least one major criterion. Uncertain malignant potential: at least one minor criterion. Benign: Absence of major and minor criteria.

Major criteria	Minor criteria
Mitoses >5 per 10 mm^2 ^(50 high power fields of 0.2 mm^2^)	Large size (>100 m and/or >200 g)
Atypical mitoses	Necrosis
Venous invasion	Capsular invasion
	Sinusoidal invasion

**Table 3:** Reticulin algorithm.

Criteria
Altered reticulin framework in association with one of the following features indicates malignancy:
Mitotic count >5 per 10mm^2^ (50 high power fields of 0.2 mm^2^)
Tumor necrosis
Vascular invasion

**Table 4:** Helsinki scoring system. Score 0 to 8.5: Benign Score >8.5: Malignant Score >17: Adverse prognosis

Parameter	Score
Mitoses >5 per 10 mm^2^ (50 high power fields of 0.2 mm^2^)	3
Necrosis	5
Ki-67 proliferation index (%)	Numeric value of the Ki-67 from the highest proliferative area

**Figure 5:**
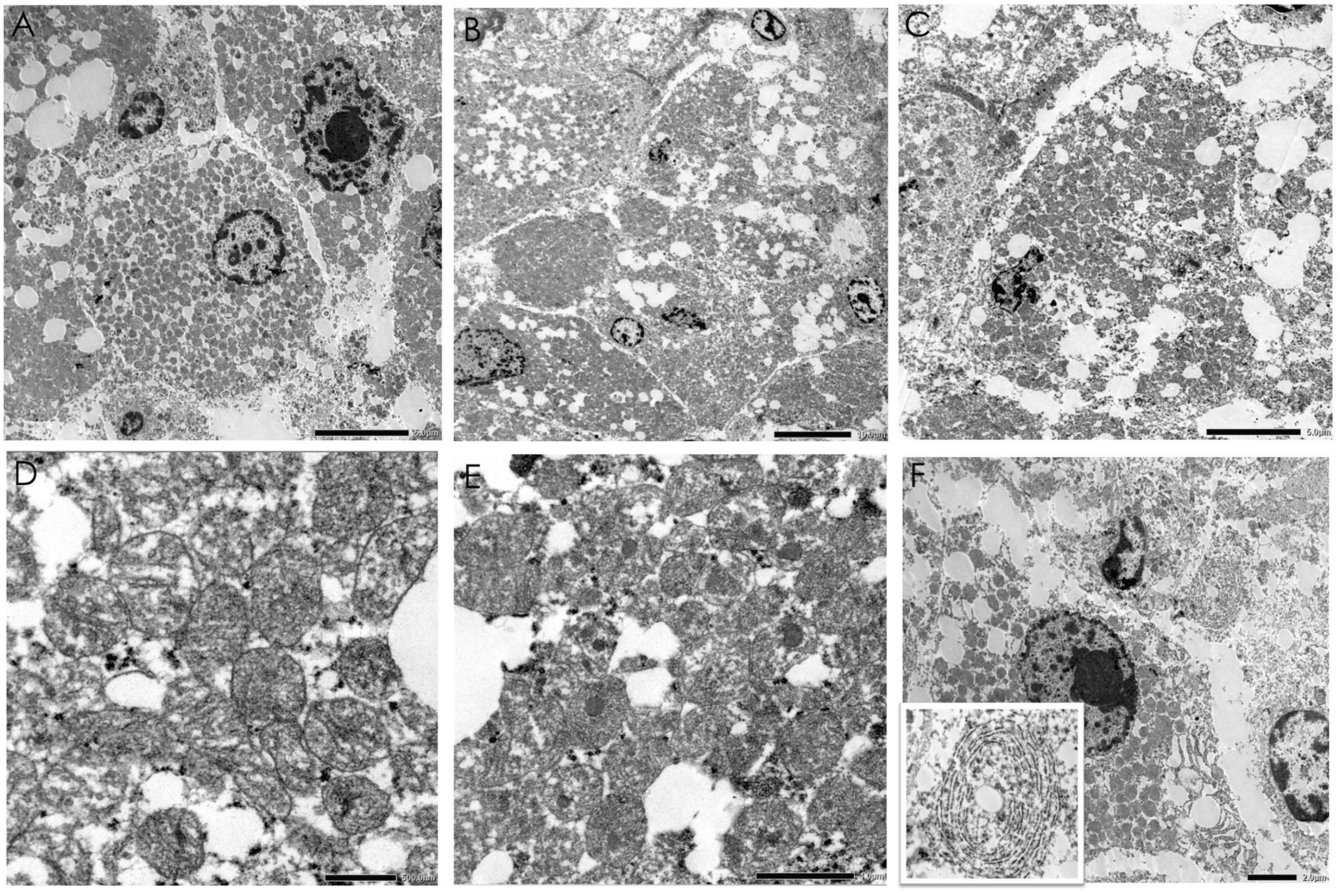
Electron microscopy of oncocytic ACC. (A) Tumor cells with nuclear irregularities (×1,500); (B) tightly packed mitochondria and lipid vacuoles (×600); (C) (×1,500); (D) mitochondria with tubular and tubulovesicular cristae (×12,000); (E) and some with electron-dense inclusion (×8,000); (F) endoplasmic reticulum forming whorls (2,000×). Inset: higher magnification of smooth endoplasmic reticulum (×12,000).

The formalin-fixed paraffin-embedded (FFPE) tissues were prepared for electron microscopy. The nuclei showed irregularities with invaginations and some with large nucleoli (Figure 5A). The oncocytic nature of the tumor is proven by the diffuse, abundant, tightly packed mitochondria representing the main cytoplasmic organelle (Figure 5A-5C). The mitochondria are circular to rod-shaped, and some are irregularly shaped. The cristae are mostly tubular (Figure 5D). A single-round, dense inclusion is present in some of the mitochondrial matrix (Figure 5E). Besides mitochondria, smooth endoplasmic reticulum in whorls and few in stacks, and lipid vacuoles are seen (Figure 5F).

A metastatic workup using CT scans revealed the presence of pulmonary and liver metastases. The patient underwent three cycles of chemotherapy with the etoposide, doxorubicin, and cisplatin (EDP) protocol. The response monitoring plan is a chest CT scan and whole abdominal CT scan every three months, and a fluorodeoxyglucose (FDG)-positron emission tomography (PET/CT) at the six-month interval as a follow-up strategy.

## Discussion

In the WHO classification of tumors, oncocytic ACC is defined by the neoplastic oncocytic cells comprising at least 90% of the entire tumor [1]. Fifty-six reported cases of this variant of ACC are documented. The tumor has a slight female predominance and no age predilection. It occurs more frequently on the left side with a left-to-right side ratio of 1.6 to 1 and no reported bilateral involvement. The recorded median size is 10 mm, with the largest dimension of 285 mm [2]. 

These tumors are non-functioning and manifest as an incidental mass or pain in half of the cases [2]. There are various imaging techniques used to evaluate tumors, with different characteristics indicating malignancy. The ultrasound features indicative of a primary malignancy can be a small (less than 6 cm), homogenous lesion, or a larger tumor with a heterogenous texture and scattered echopenic areas of hemorrhage and necrosis. A CT scan will further characterize and classify the mass as malignant with the following results: a large mass measuring greater than 4 cm, with high Hounsfield density (>10 Hounsfield units), central areas of low attenuation, presence of calcification, and liver or regional lymph node metastases, including left renal vein or inferior vena cava extension. Lastly, magnetic resonance imaging (MRI) studies of ACC will frequently be heterogeneous and hyperintense on both T1- and T2-weighted sequences, with high signal intensity on T2-weighted images and heterogenous enhancement on gadolinium-enhanced T1-weighted sequences [3].

On the other hand, a quarter of the tumors are functional with the production of sex hormones, while the other quarter may show cortisol, aldosterone, or multihormonal production [2]. Besides clinical manifestations, the European Network for the Study of Adrenal Tumors (ENSAT) recommended screening tests to assess hormone excess. This includes measurement of plasma or urinary metanephrines and a 1-mg overnight dexamethasone suppression test. The clinical presentation, imaging findings, and other pertinent ancillary studies guide the clinician in evaluating the risk of malignancy and surgical plans. An open adrenalectomy for masses with suspicious features of malignancy is recommended, and an adrenal tissue biopsy is not advocated [4].

Macroscopically, the tumors are circumscribed, encapsulated, large, and rounded, with tan or brown cut surfaces and areas of hemorrhage and necrosis. Microscopic appearance will show diffuse sheets and trabecular architecture of neoplastic cells with abundant deeply eosinophilic granular cytoplasm and nuclei with high-grade and bizarre atypia [5]. These morphologic characteristics were found in the case at hand. As a result, the differential diagnoses were limited to an adrenal cortical adenoma, pheochromocytoma, and metastatic carcinoma (grade IV clear cell renal cell carcinoma, chromophobe renal cell carcinoma, hepatocellular carcinoma).

Several multiparameter scoring systems are used in ACC, but the Lin-Weiss-Bisceglia system is recommended in oncocytic adrenal neoplasms to avoid overcalling malignancy. This eliminates three of the Weiss criteria, which are already features of oncocytic tumors, namely: having a high nuclear grade, less than 25% clear cells, and a diffuse architecture [2]. The present case satisfies all major and minor criteria of the LWB scoring system, proving its malignancy and effectively ruling out an adenoma.

SF-1 is the most specific biomarker to show evidence of adrenal cortical origin. As an alternative, immunohistochemistry with Melan-A, synaptophysin, alpha-inhibin, and calretinin are used [1]. The present case had positive expression in melan-A and synaptophysin and negative expression in pancytokeratin, chromogranin, and hep-Par1. This profile rules out a metastatic carcinoma and an adrenal medullary tumor, specifically, pheochromocytoma. Although ACC is a malignant epithelial tumor, pancytokeratin expression is usually variable, and epithelial membrane antigen (EMA) and carcinoembryonic antigen (CEA) are negative [1]. The morphologic and immunohistochemistry profile of the current case supports an adrenal cortical carcinoma.

An electron microscopy study was done to show evidence of its oncocytic nature. The polygonal neoplastic cells are predominantly packed by variably sized, round, ovoid, irregularly-shaped mitochondria, which is similar to other ultrastructural reports of oncocytic adrenal cortical neoplasms (Figure 5) [6-9]. Additionally, the current case had the same spherical, dense intramitochondrial inclusion documented in the investigations of El-Naggar et al. [7] and Bisceglia et al. [8]. Immunohistochemistry with antimitochondrial antibody (AMA) mes-13 can be an alternative method of documenting mitochondria [8-9]. However, the non-oncocytic adrenal cortical cells would likewise have increased mitochondria. Hence, there is a strict characteristic strong, diffuse, finely granular cytoplasmic expression necessary to classify an oncocytic differentiation using this immunohistochemistry stain [8]. Tumor classification is part of the 23 core elements for reporting ACC, according to the International Collaboration on Cancer Reporting (ICCR). ICCR also lists specimen integrity, greatest dimension, weight, extent of invasion, architecture, percentage of lipid-rich cells, capsular invasion, lymphatic invasion, vascular invasion, atypical mitotic figures, coagulative necrosis, nuclear grade, mitotic count, Ki67 proliferative index, margin status, lymph node status, and pathological stage [10]. These 23 core elements show evidence of validity in the management, staging, and prognosis of adrenocortical carcinomas.

Metastases only occur in 13% of the reported oncocytic ACC cases. Furthermore, this variant has the best prognosis among others, with a median survival of 60 months - almost twice the median survival of the conventional ACC in adults [2]. Therefore, the classification of these tumors would have clinical importance.

Complete surgical resection remains the only potential curative treatment strategy. However, chemotherapeutic adjuvant treatment with mitotane showed beneficial outcomes [11]. Unfortunately, this is not available in the Philippines. As an alternative, a combination of cisplatin, etoposide, and doxorubicin was offered to the patient.

## Conclusions

Oncocytic ACC is a relatively rare malignant neoplasm with a good prognosis. The morphologic features and immunohistochemical evidence of adrenal cortical differentiation, together with the appropriate multiparameter scoring system and proliferative index, are necessary for the investigation of an ACC. Oncocytic adrenal cortical neoplasms warrant evaluation with the Lin-Weiss-Bisceglia system, Helsinki scoring system, and/or reticulin algorithm to classify malignancy and aid in prognostication. A confirmation with electron microscopic studies or an anti-mitochondrial immunohistochemistry study is recommended to document oncocytic differentiation. Advanced studies, particularly molecular profiling of these tumors, are recommended to provide more insights and further improve clinical outcomes. Complete surgical resection is the mainstay treatment, but adjuvant chemotherapy with Mitotane may prolong recurrence-free survival.
